# Principles for delivery of youth lay counsellor programs: Lessons from field experiences

**DOI:** 10.7189/jogh.12.03047

**Published:** 2022-07-25

**Authors:** Merrian J Brooks, Nicola Willis, Rhulani Beji-Chauke, Ontibile Tshume, Onkemetse Phoi, Elizabeth Lowenthal, Dixon Chibanda, Rashida A Ferrand

**Affiliations:** 1 Children’s Hospital of Philadelphia, Philadelphia, Pennsylvania, USA; 2 University of Pennsylvania, Perelman School of Medicine, Philadelphia, Philadelphia, Pennsylvania, USA; 3 Botswana UPENN Partnership, Gaborone, Botswana; 4 Zvandiri, Harare, Zimbabwe; 5 Friendship Bench Zimbabwe, Harare, Zimbabwe; 6 Botswana Baylor Children’s Clinic, Gaborone, Botswana; 7 London School of Hygiene and Tropical Medicine, London, UK; 8 Biomedical Research and Training Institute, Harare, Zimbabwe

**Figure:**
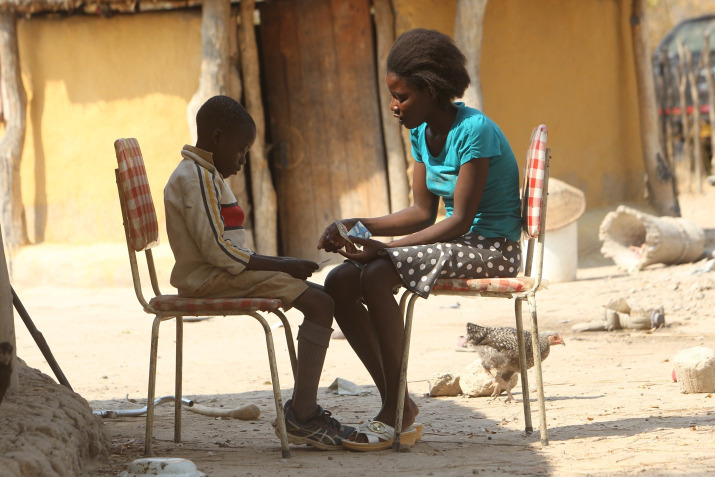
Photo: CATS counselling her client in rural Zimbabwe. Photography credit: Tsvangirai Mukwazhi, used with permission (Commissioned by author NW).

There is accumulating evidence that lay counsellors can effectively provide structured, evidence-based counselling interventions to improve mental health outcomes [1-4]. Lay counsellors help alleviate overburdened health systems that have limited numbers of mental health professionals through 'task-shifting,' a process of referring straightforward, or common mental health illness to lay workers who have received counselling training from mental health professionals [5].

Lay counsellor-based programs have been scaled-up within large-scale projects such as those in India’s mental health program the Healthy Activity Program where the contact coverage for most common mental disorders and substance abuse is very low and budgetary allocations for mental health care minimal [1,6], and in Zimbabwe’s Friendship Bench program that successfully trialled a task-shifting intervention for common mental disorders delivered by trained and supervised lay health workers [7]. Additionally, non-profit organizations in Zimbabwe such as Zvandiri, a model of peer-led differentiated service delivery, delivers integrated HIV, mental health and protection services for children, adolescents and young people living with HIV across the health facilities and communities using trained, mentored youth lay counsellors (YLCs) for children and adolescents living with HIV and common mental disorders called Community Adolescent Treatment Supporters (CATS) within government health facilities across the country. Zvandiri CATS’ engagement with youth living with HIV has helped to improve adherence to antiretroviral therapy, rates of viral suppression and decreased the prevalence of common mental disorders in this population [8].

Interventions incorporating HIV/AIDS peer education have had a positive impact on outcomes such as HIV risk behaviours [9,10]. As opposed to hierarchical client/youth counsellor frameworks, peer educators influence change through a horizontal process of peer-to-peer communication and collective decision-making. While a peer educator is specifically a person of equal standing with a patient or who belongs to the same age-, societal- or other characteristic group, who helps a patient effect change in their knowledge, attitudes, beliefs, or behaviours by modelling such behaviours [11], we designated lay counsellors as those aged 19 to 27 specifically trained to provide evidence-based counselling modalities and therapies.

When a lay counselling program, such as the Zvandiri program, utilizes youth as lay counsellors there are additional dynamics to consider. Although the youth are well suited to provide support to their peers [12], there are important contexts to be considered when working with YLCs. Youth may have fewer life skills and coping skills, and less social support than adults. Some youth may also be managing their clients’ problems in the context of their own stressors including high youth unemployment or under-education.

We propose seven principles for incorporating YLCs in mental health programmes. These are rooted in program and research evidence from the Zvandiri program [13,14], discussions with youth lay counsellors aged 19 to 27 (Brooks 2020, unpublished data) and from experiences from YLC programmes including a pilot YLC program in Botswana, and two youth centred lay counselling programs based on the Friendship Bench model (Youth Friendship Bench (YouFB)) in Zimbabwe.

## PRINCIPLES FOR YOUTH LAY COUNSELLOR PROGRAMS

### YLC should receive appropriate structured supervision from mental health professionals

This type of supervision should include immediate support for lay counsellors attending to a client in a mental health emergency or crisis [15]. During the pilot phase of the youth-delivered problem-solving therapy in Botswana, there was direct on-site professional support for lay counsellors from a program partner trained in mental health first aid who could escalate complex cases to a mental health professional (ie, a psychologist, social worker, or physician with mental health training on a rotating schedule). YLCs under the YouFB and in the Zvandiri programs in Zimbabwe usually receive ongoing supervision from clinical psychologists. [5]

The second type of supervision, case review with professionals, is commonly used by mental health professionals in many settings. Mental health professionals (eg, social workers, psychologists, psychiatrics, therapist), regularly review complex (and regular) clients in conjunction with the lay counsellors in a group setting or review taped sessions with each individual lay counsellor. This supervision can even be done virtually, as has been done for the Zvandiri YLCs during the SARS-CoV-2 pandemic. Virtual supervision can work well for programs where the team is in multiple locations, or where due to a limited number of local experts, remote expert support is needed. This type of supervision ensures that all clients, including those whose conditions YLCs may not adequately address, get the assistance they need [16]. Lay counsellors from the YouFB in Zimbabwe described cases and types of difficulties their clients were managing that were not included in the training manual. These include situations such as problems resulting from sexual activity, decisions related to abortions, and discrimination related to homosexuality [14]. These regular supervision sessions allowed lay counsellors to increase their confidence and skills to approach emerging challenge and expanded their skills in dealing with such issues.

### Establish clear working roles and responsibilities for YLC

Because YLCs may have diverse prior training and experiences, it is critical to clearly define the boundaries of their professional responsibilities. For example, in the YLC program piloted in Botswana, some lay counsellors had mental health training that exceeded the program's requirements. Therefore, we established protocols to manage situations such as 'suicidal ideation,' 'medically unwell,' 'unsafe home environment,' etc. Youth counsellor programs should emphasize best practices when engaging with minors such as facilitating the reporting of child abuse and limits to confidentiality. These best practices and professional skills ought to be integrated by a professional team before engaging lay counsellors and throughout a lay counsellor program [17]. Regardless of whether the YLCs felt that they could handle situations beyond their defined YLC role, all YLCs had to follow protocols for escalation to protect clients and YLCs alike [18].

When not engaged in peer counselling, YLCs might also serve as patient navigators, educators, and social service coordinators. These other identities can make it difficult for YLCs to focus on delivering the evidence-based therapy when they are serving in the YLC role. Therefore, health programs should clearly specify roles, protect time and space for the counselling work. Additionally, health programs should guide YLCs to follow set protocols for referral and escalation to a mental health professional.

### Establish clear community roles and personal boundaries

Lay counsellors often reside within the communities they serve. Additionally, for those who have never had professional job experiences, the young persons’ familiarity with professional norms should not be assumed. Issues of confidentiality and professional boundaries need to be stressed in YLC training sessions and reinforced during supervision discussions. YLCs in Botswana asked for guidance on scenarios, such as personal feelings between counsellors and clients, intimate relationships between other team members, and maintaining confidentiality and how to respond during chance community encounters with clients. Like the processes used to establish and reinforce roles and responsibilities for YLCs, ongoing training and supervision regarding maintaining personal boundaries is helpful.

### Define parameters of onset and conclusion of service

At the conclusion of services, YLCs should be truly integrated within the health care system and care pathway to facilitate transitions if a counselling program ends, the YLC ages out of the program, or the client needs to be referred to a higher level of care. YLCs should be assisted to navigate the sometimes challenging and emotional task of establishing new boundaries with former clients. This can be done by creating a referral system for those cases where further management is needed. A ‘handover plan,’ which includes a list of referral resources, might also be helpful for programs. Define parameters of onset and conclusion of service helps youth lay counsellors have a sense of closure and move on without feeling as if they are abandoning their clients, thus creating a sense of obligation to continue to provide support outside the professional boundaries and support systems [18].

### Provide high quality training for YLC

While lay counsellors are not professionally trained, health programs should appropriately invest in the training and mentorship of YLCs. For example, an initial two weeks-long training session in Problem-Solving Therapy may ensure adequate training in the type of counselling they are expected to deliver. Trainings should be protocolized and iterative to meet the needs of the trainees over time. The training modality and training tools should be appropriate for the established educational level for the YLC in a specific program. For instance, in the Botswana program, youth who had recently completed secondary school were eligible to serve as YLCs. Therefore, they needed foundational training. Conversely, the YLC in the YouFB program in Zimbabwe had at least tertiary education; their training needs would therefore differ from that we offered. Additionally, training sessions should include opportunities to practice information imparted during theoretical sessions, and assessments to demonstrate competencies such as mock cases and role playing. Furthermore, training competencies should be reinforced through continuous supervision structures as outlined above [19].

### Directly invest in lay counsellors’ mental health

The protection of YLCs’ mental health is essential to the success of YLC programs. YouFB Zimbabwe counsellors stated that they were ‘carrying an emotional burden’ from problems brought up during counselling sessions with clients [14,18]; a burden often compounded by the YLCs own unresolved problems. The YLCs further mentioned that they needed therapy sessions after having sessions with clients [14]. The investment in YLCs’ mental health can include support groups for lay counsellors, personal check-ins during periodic one-on-one meetings with supervisors, or access to free or subsidized mental health resources [18].

### Appropriately compensate YLC as valuable members of the health care team

YLCs should be reimbursed in a way that is commensurate with their roles and responsibilities in their local contexts [20]. Although the amount of financial compensation will differ by context, Institutional Review Boards, donors, and government budgets must support the lay counsellors by acknowledging their contribution to the health care system. Furthermore, YLCs are often in the process of establishing their professional lives, getting married and having children, and attending higher education programs. Youth involvement in lay counsellor programs affords them an opportunity for financial independence as well as a springboard to future opportunities. Although our work in Botswana showed that YLCs have a lot of gain from being lay counsellors, temporary positions are sometimes at odds with other opportunities for personal advancement. At the request of the YLCs, the Botswana program provided letters of recommendation and professional development sessions for YLCs on topics such as updating a resume, interviewing for a job, and other job skills. YouFB counsellors in Zimbabwe reported gaining mentorship, problem-solving skills, emotional regulation, public speaking skills, and self-care skills [14]. These non-financial modes of compensation allowed us to indirectly contribute to their futures albeit in a limited manner. Alternative methods to support lay counsellors include raising funds for scholarships to further their education, or establishing relationships with NGOs and other research partners to engage YLCs in employment or professional development [20]. Ideally, youth counsellors could be integrated into the local system, as they have been in the Zvandiri program where YLCs have now been trained and employed by Ministry of Health and Child Care in Zimbabwe.

## CONCLUSION

YLCs, provide meaningful and effective services, particularly in resource-stretched health settings. Furthermore, they may arguably be the best people to provide mental health support for other youth because of their shared lived experiences and relatability [12,13]. We must be thoughtful about how we support YLCs, like any other valuable team members, so that they operate under optimal conditions so that they serve the community with their needs and circumstances taken into consideration.
